# Diffuse Idiopathic Skeletal Hyperostosis: Persistent Sore Throat and Dysphagia in an Elderly Smoker Male

**DOI:** 10.1155/2017/2567672

**Published:** 2017-09-14

**Authors:** Ana Goico-Alburquerque, Beenish Zulfiqar, Ranae Antoine, Mohammed Samee

**Affiliations:** Internal Medicine Department, Advocate Illinois Masonic Medical Center, Chicago, IL, USA

## Abstract

Diffuse idiopathic skeletal hyperostosis (DISH) is rarely symptomatic. However, it can present with dyspnea, hoarseness, dysphagia, and stridor. An 80-year-old chronic smoker male presented with 6-month history of sore throat and progressive dysphagia. Computed tomography of the neck revealed bulky anterior bridging syndesmophytes along the anterior aspect of the cervical spine and facet effusion involving four contiguous vertebrae consistent with DISH. Dysphagia secondary to DISH was diagnosed. Fiberoptic laryngoscopy showed bilateral vocal cord paralysis. Patient's airway became compromised requiring tracheostomy tube placement. After discussion of therapeutic options, patient agreed on a percutaneous endoscopic gastrostomy tube insertion for nutritional support. Osteophytectomy was left to be discussed further.

## 1. Introduction

Sore throat and oropharyngeal dysphagia constitutes complaints fairly encountered in the elderly population [1]. Diffuse idiopathic skeletal hyperostosis (DISH), also known as Forestier's disease, was first described by Forestier and Rotes-Querol in 1950 [2]. It is an idiopathic systemic disorder primarily affecting the axial skeleton characterized by ossification of anterolateral vertebral ligaments and anterior osteophyte formation along the spinal column [2]. Although it is mostly asymptomatic, progression of anterior cervical hyperostosis can cause symptoms frequently seen in clinical practice such as dysphagia, hoarseness, and airway obstruction [3]. We describe a case of an 80-year-old man with heavy smoking history who presented with painful swallowing and weight loss secondary to DISH.

## 2. Case Presentation

An 80-year-old African-American male presented with the main complaint of persistent sore throat and progressive odynophagia and dysphagia to solids and liquids over the past six months along with nonproductive cough, difficulty breathing, and a 20 lbs weight loss that developed over the course of one month. Pertinent past medical history included hypertension, type 2 diabetes mellitus, and a 10-pack-year smoking history. Patient was found to be tachycardic (120 bpm), saturating 92% on 2 L of oxygen by nasal cannula. On exam, the patient looked cachectic, in mild distress, with a notable hoarse voice. No visible masses were seen in oropharynx nor palpated on the neck. Significant inspiratory stridor was auscultated at the lung apex with no other adventitious lung sounds. The rest of examination was unremarkable. Laboratory work was only significant for normocytic anemia with hemoglobin of 10.5 g/dl.

**Figure 1:**
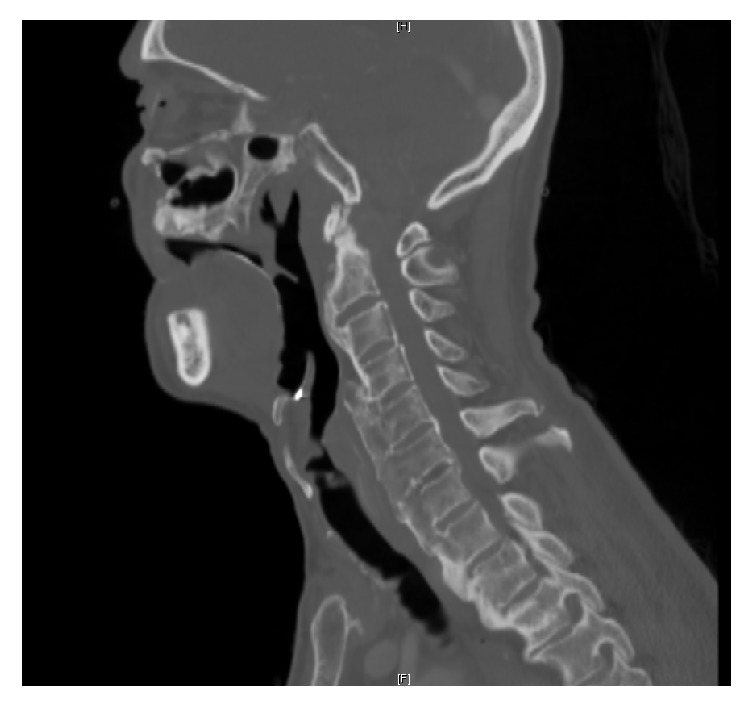
CT scan of the cervical spine reveals continuous and irregular hyperostosis alongside the anterior aspect of the cervical spine consistent with DISH.

Planned barium swallow was deferred due to minimal aspiration on videofluoroscopy. Computed tomography (CT) neck was obtained revealing bulky anterior bridging syndesmophytes along the anterior aspect of the cervical spine and facet effusion most marked at the C2-C3 and C3-C4 vertebrae consistent with DISH (Figure 1). Fiberoptic laryngoscopy revealed a fixed left vocal cord and very minimal abduction of the right vocal cord. The patient developed a severely compromised airway which subsequently required placement of a tracheostomy tube. After extensive discussion with the patient, the power of attorney, and involved physicians, the decision was made to insert a percutaneous endoscopic gastrostomy tube for fulfillment of nutritional needs. The patient tolerated both procedures and the recovery period. The option for osteophytectomy was left to be discussed with his primary care physician following discharge. The patient was discharged to a long-term acute care facility, where he received continued care.

## 3. Discussion

DISH is a noninflammatory condition characterized by calcification and ossification of soft tissues, mainly ligaments and enthesis recognized as a form of degenerative arthritis or osteoarthritis [2]. A characteristic radiographic feature of DISH is the presence of hyperostosis of the cortex along the anterior surface of the vertebrae which can gradually elongate and grow across the disc space [4, 5].

Most authors agree that the course of DISH is typically benign and consider the skeletal abnormalities as radiographic findings rather than a disease [6, 7]. Whether the condition itself is a cause of significant pain and whether it is a true disease entity still remain controversial [8]. Studies show DISH to be most prevalent in developed countries, although this apparent predominance might be due to the more frequent use of radiological examinations in these countries than in others [9]. The condition is unequally distributed between males and females (in a ratio of ~2 : 1), and its prevalence rapidly increases with age, most commonly after fifth or sixth decades of life [5, 10, 11]. American blacks may have a lower prevalence of DISH than whites as with our patient [12].

Some authors state increasing evidence indicating DISH as the underlying cause of pathological conditions. Furthermore, complaints among those with this disorder will vary by the site of spinal involvement [5, 13]. Patients with cervical osteophytes usually will present with complaints of dysphagia and airway compromise, with levels C2, C3, C4, and C5 most implicated in symptomatic disease such as the present case [14]. Dysphagia is the second most common symptom of DISH after thoracic spinal pain and is reported in 15 to 25 percent of patients [13, 15]. Large cervical osteophytes can cause swallowing disorders from esophageal compression, localized inflammation or ulceration, restricted motion of the epiglottis and larynx, and narrowing of the pharyngeal wall [16].

Interestingly, there is no conclusive correlation between the osteophyte size and symptoms. However, older age has been related to more severe symptoms [17]. Some reports have found that approximately 70% patients will seek medical attention after six months of initiation of dysphagia as with our patient [14]. An explanation for this would be that initially patients will try to mechanically alter their diet and will cope to swallow food with some extra effort and often will seek advice from a physician when the problem becomes difficult to manage. Because life expectancy is increasing, the prevalence of dysphagia secondary to DISH may also increase in the coming decades [14].

The exact mechanism of DISH remains unknown as current literature is not considered to be robust. Various hypotheses include mechanical factors, diet, metabolic conditions, and environmental exposures [5]. DISH is associated with a metabolic syndrome. Clinical observations have found a positive correlation with obesity, type two diabetes, and hypertension, among others [5, 18]. Of all potential factors, type two diabetes and obesity correspond with a hyperinsulinemic state which is hypothesized to increase several growth factors and inflammatory mediators, which then leads to proliferation of osteoblasts, chondrogenesis, and possibly subsequent ossification in ligaments [19, 20].

Formal diagnosis of DISH in patients presenting with dysphagia should involve imaging studies. To diagnose DISH, plain radiographs of the cervical spine should be consistent with Resnick and Niwayama criteria (Table 1) [21]. CT and magnetic resonance imaging (MRI) of the spine are also useful and provide additional information, such as assessment of affected soft tissue and other important structures [22]. More invasive diagnostic tests such as barium swallow, nasopharyngoscopy, videofluoroscopy, and laryngoscopy are also useful for visualizing potential mechanical obstruction or to rule out malignancy [22, 23].

Some sources suggest that initial treatment should include conservative measures including speech and swallow therapy, diet modification, physical activity, and weight reduction, which should help to reduce hyperinsulinemia which has been thought to have a role in the pathogenesis of DISH. Anti-inflammatory medication and steroids have also shown some benefit. Others advocate that these methods may only offer temporary relief and surgery should be envisioned as the definitive solution in patients affected by dysphagia, airway compromise, and substantial weight loss. This can include tracheostomy and gastric tube placement for nutritional support or osteophytectomy which is considered to be highly successful when conservative methods fail [22, 23]. Several authors have reported cases of dysphagia including one with bilateral vocal fold paralysis secondary to DISH where tracheotomy and cervical osteophytectomy have successfully been performed, reporting marked improvement in swallowing function and speaking ability [14, 23–25]. However, recognizing the limited data regarding this condition, studies assessing the efficacy of both strategies to treat dysphagia and/or airway obstruction in the presence of DISH are lacking. Interestingly, postsurgical recurrence of osteophytes in DISH has also been reported [26].

## 4. Conclusion

DISH is a relatively overlooked disease that is uncommonly symptomatic in older people. It is a condition that has not been fully elucidated and is an underappreciated cause of dysphagia/airway obstruction in this population. Its diagnostic evaluation is mainly radiological. Further studies are necessary to determine the pathogenesis as well as long-term efficient therapeutic approaches. In a chronic elderly smoker presenting with progressive dysphagia, it is essential to rule out malignancy. However, it is also necessary to keep in mind rare diagnosis like DISH which requires imaging and prompt surgical evaluation. The present data supports the high incidence of DISH according to our patient's comorbidities, although no association was found between DISH and heavy tobacco use.

## Figures and Tables

**Table 1:** Criteria to diagnose DISH according to Resnick and Niwayama [21].

(i) Calcification and ossification along the anterior surface of four contiguous vertebrae
(ii) Preserved intervertebral disc height
(iii) Absence of apophyseal joint ankylosis and sacroiliac joint sclerosis, erosion, or intraarticular bony effusion

## References

[1] Sura L., Madhavan A., Carnaby G., Crary M. A. (2012). Dysphagia in the elderly: Management and nutritional considerations. *Clinical Interventions in Aging*.

[2] Forestier J., Rotes-Querol J. (1950). Senile ankylosing hyperostosis of the spine. *Annals of the rheumatic diseases*.

[3] Pulcherio J. O. B., Velasco C. M. M. D. O., Machado R. S., de Souza W. N., de Menezes D. R. (2014). Forestier's disease and its implications in otolaryngology: literature review. *Brazilian Journal of Otorhinolaryngology*.

[4] Giuffra V., Giusiani S., Fornaciari A., Villari N., Vitiello A., Fornaciari G. (2010). Diffuse idiopathic skeletal hyperostosis in the Medici, Grand Dukes of Florence (XVI century). *European Spine Journal*.

[5] Mader R., Verlaan J.-J., Buskila D. (2013). Diffuse idiopathic skeletal hyperostosis: Clinical features and pathogenic mechanisms. *Nature Reviews Rheumatology*.

[6] Beyeler C. H., Schlapbach P., Gerber N. J. (1992). Diffuse idiopathic skeletal hyperostosis (dish) of the elbow: A cause of elbow pain? a controlled study. *Rheumatology*.

[7] Fahrer H., Barandun R., Gerber N. J., Friederich N. F., Burkhardt B., Weisman M. H. (1989). Pelvic manifestations of diffuse idiopathic skeletal hyperostosis (DISH): are they clinically relevant?. *Rheumatology International*.

[8] Hutton C. (1989). Dish...a state not a disease?. *Rheumatology*.

[9] Cassim B., Mody G. M., Rubin D. L. (1990). The prevalence of diffuse idiopathic skeletal hyperostosis in African Blacks. *British Journal of Rheumatology*.

[10] Kiss C., O'Neill T. W., Mituszova M., Szilágyi M., Donáth J., Poór G. Y. (2002). Prevalence of diffuse idiopathic skeletal hyperostosis in Budapest, Hungary [5]. *Rheumatology*.

[11] Westerveld L. A., Quarles Van Ufford H. M. E., Verlaan J.-J., Oner F. C. (2008). The prevalence of diffuse idiopathic skeletal hyperostosis in an outpatient population in the Netherlands. *Journal of Rheumatology*.

[12] Utsinger PD. (1985). Diffuse idiopathic skeletal hyperostosis. *Rheumatic Disease Clinics*.

[13] Mata S., Fortin P. R., Fitzcharles M.-A. (1997). A controlled study of diffuse idiopathic skeletal hyperostosis: Clinical features and functional status. *Medicine*.

[14] Verlaan J.-J., Boswijk P. F. E., De Ru J. A., Dhert W. J. A., Oner F. C. (2011). Diffuse idiopathic skeletal hyperostosis of the cervical spine: an underestimated cause of dysphagia and airway obstruction. *Spine Journal*.

[15] Castellano D. M., Sinacori J. T., Karakla D. W. (2006). Stridor and dysphagia in diffuse idiopathic skeletal hyperostosis (DISH). *Laryngoscope*.

[16] Oppenlander M. E., Orringer D. A., La Marca F. (2009). Dysphagia due to anterior cervical hyperosteophytosis. *Surgical Neurology*.

[17] Seidler T. O., Pèrez Àlvarez J. C., Wonneberger K., Hacki T. (2009). Dysphagia caused by ventral osteophytes of the cervical spine: clinical and radiographic findings. *European Archives of Oto-Rhino-Laryngology*.

[18] Mader R., Lavi I. (2009). Diabetes mellitus and hypertension as risk factors for early diffuse idiopathic skeletal hyperostosis (DISH). *Osteoarthritis and Cartilage*.

[19] Pillai S., Littlejohn G. (2014). Metabolic factors in diffuse idiopathic skeletal hyperostosis - A review of clinical data. *Open Rheumatology Journal*.

[20] Akune T., Ogata N., Seichi A., Ohnishi I., Nakamura K., Kawaguchi H. (2001). Insulin secretory response is positively associated with the extent of ossification of the posterior longitudinal ligament of the spine. *Journal of Bone and Joint Surgery - Series A*.

[21] Resnick D., Niwayama G. (1976). Radiographic and pathologic features of spinal involvement in diffuse idiopathic skeletal hyperostosis (DISH). *Radiology*.

[22] Seidler T., Alvarez J., Wonneberger K. (2009). Dysphagia caused by a ventral osteophytes of the cervical spine: clinical and radiographic findings. *European Archives of Oto-Rhino-Laryngology*.

[23] Lecerf P., Malard O. (2010). How to diagnose and treat symptomatic anterior cervical osteophytes?. *European Annals of Otorhinolaryngology, Head and Neck Diseases*.

[24] von der Hoeh N. H., Voelker A., Jarvers J. S., Gulow J., Heyde C. E. (2015). Results after the surgical treatment of anterior cervical hyperostosis causing dysphagia. *European Spine Journal*.

[25] Allensworth J. J., O'Dell K. D., Schindler J. S. (2017). Bilateral vocal fold paralysis and dysphagia secondary to diffuse idiopathic skeletal hyperostosis. *Head and Neck*.

[26] Miyamoto K., Sugiyama S., Hosoe H., Iinuma N., Suzuki Y., Shimizu K. (2009). Postsurgical recurrence of osteophytes causing dysphagia in patients with diffuse idiopathic skeletal hyperostosis. *European Spine Journal*.

